# Prediction of Split Renal Function Improvement After Renal Artery Stenting by Blood Oxygen Level–Dependent Magnetic Resonance Imaging

**DOI:** 10.3389/fcvm.2022.793777

**Published:** 2022-02-28

**Authors:** Zhiyong Lin, Bihui Zhang, Letao Lin, Rui Wang, Guochen Niu, Ziguang Yan, Yinghua Zou, Xiaoqiang Tong, Jianxing Qiu, Min Yang

**Affiliations:** ^1^Department of Interventional Radiology and Vascular Surgery, Peking University First Hospital, Beijing, China; ^2^Department of Radiology, Peking University First Hospital, Beijing, China; ^3^State Key Laboratory of Oncology in South China, Collaborative Innovation Center for Cancer Medicine, Sun Yat-sen University Cancer Center, Guangzhou, China; ^4^Minimally Invasive Interventional Division, Sun Yat-sen University Cancer Center, Guangzhou, China

**Keywords:** atherosclerotic renal artery stenosis, renovascular stenting, renal artery stenting, blood oxygen level-dependent magnetic resonance imaging, prediction

## Abstract

**Background:**

The discrepancy between the high technical success rate and the relatively low clinical response rate of renal artery stenting (RAS) raises the importance to identify atherosclerotic renal artery stenosis (ARAS) patients who are most likely to benefit from RAS. This study aimed to investigate the feasibility and accuracy of blood oxygen level-dependent magnetic resonance imaging (BOLD-MRI) in predicting split renal function (SRF) improvement after RAS in patients with ARAS.

**Methods:**

Thirty patients with severe ARAS who were treated with RAS were enrolled. Baseline cortical and medullary R2* values of each kidney were measured by BOLD-MRI, and each patient’s SRF was evaluated by nuclear renal dynamic imaging at baseline and 1-month follow-up.

**Results:**

In total, 35 severe stenotic renal arteries of the 30 patients were analyzed. At 1-month follow-up, 34 kidneys (97.1%) of severe ARAS had acquired SRF. SRF improved in 12 kidneys of 10 patients. The cortical R2* and medullary R2* values in the SRF improvement kidneys were higher than those in the non-improvement kidneys (*P* ≤ 0.001). The area under the curve of medullary R2* was 0.879 (95% confidence interval [CI] 0.736–1.000). A medullary R2* value ≥29.1 s^–1^ was noted to provide good sensitivity (0.833, 95% CI 0.552–0.970) and specificity (0.864, 95% CI 0.667–0.953) in predicting SRF improvement. Medullary R2* value was the only independent predictor of SRF improvement in multivariable analysis (*P* = 0.034, OR 3.017, 95%CI 1.089–8.358).

**Conclusion:**

This study showed that a BOLD-MRI medullary R2* value ≥29.1 s^–1^ was an excellent predictor of SRF improvement in patients with severe ARAS who underwent renal artery stenting.

## Introduction

Atherosclerotic renal artery stenosis (ARAS) is the most common cause of secondary hypertension, with severe stenosis (>60%) occurring in 6.8% of elderly patients (1–3). Renal artery stenting (RAS) is the preferred treatment option. Still, recent randomized trials have reported that when RAS is combined with the best medical therapy, there is no benefit regarding blood pressure, renal function, cardiovascular events, or mortality compared to medical treatment alone (4–6). However, these trials had significant design flaws, including variability in inclusion and exclusion criteria, inconsistent definitions of improvement, and differing endpoints, making the selection of patients for renal artery stenting controversial (7–9). For this reason, determining a biomarker to help predict RAS outcomes and identify patients who would best respond to RAS is of great clinical significance (8, 9).

Currently, several biomarkers are used to select patients likely to benefit from RAS, including resistance index (RI), translesional pressure gradient, renal artery fractional flow reserve, and brain natriuretic peptide (10). However, these methods are either invasive or not sufficiently accurate to be applied widely. Blood oxygen level-dependent magnetic resonance imaging (BOLD-MRI) is a non-invasive imaging technique that can assess the kidney’s regional oxygenation (11). It uses endogenous deoxygenated hemoglobin as a contrast agent and detects magnetic field disturbances caused by changes of oxygenated hemoglobin within the kidney tissue (11). The R2* score is the magnetic rate of relaxation that correlates positively with deoxyhemoglobin levels and can evaluate the severity of ischemia caused by ARAS (12, 13).

The present study evaluated the feasibility and accuracy of using BOLD-MRI to identify severe ARAS patients who might benefit from RAS in split renal function (SRF).

## Materials and Methods

### Patients

This prospective study enrolled patients with severe ARAS consecutively from May 2018 to November 2018 and was approved by the Human Investigations Committee of Peking University First Hospital (2018263). The inclusion criteria were as follows: (1) patient age was ≥40 years; (2) the degree of stenosis on the treatment side of the renal artery was ≥70% (including occlusion), and there was no definite stenosis in the branches of the renal artery; (3) Renal artery stenosis was caused by atherosclerosis, which included ➀ a minimum of 1 atherosclerosis risk factor (age ≥40, diabetes, hyperlipidemia, obesity, or long-term smoking) and ➁ a minimum of 2 imaging features of atherosclerosis (the lesion involved the ostium or proximal segment of the renal artery, eccentric stenosis or occlusion, irregular plaque, calcification, and signs of atherosclerosis in other abdominal vessels); (4) clinical symptoms related to renal artery stenosis were present, including hypertension (blood pressure >140/90 mmHg), impaired renal function (taking renal dynamic imaging as the reference standard, unilateral glomerular filtration rate (GFR) <34 ml/min), or acute pulmonary edema and unstable angina pectoris; and (5) patients signed informed consent. Exclusion criteria mainly included the following: Renal artery stenosis caused by non-atherosclerotic factors, such as Takayasu arteritis, fibromuscular dysplasia, congenital arterial malformation, restenosis after renal artery intervention, or graft RAS; severe renal dysfunction [serum creatinine >264 μmol/L (3.0 mg/dl)]; and atrophied kidney (length <7 cm). The complete inclusion and exclusion criteria are shown in the appendix. SRF was evaluated with radionuclide renal dynamic imaging and the GFRs of both kidneys were obtained at baseline and 1-month follow-up.

### Endovascular Procedures

Patients were treated by experienced vascular specialists in a catheter lab. Local anesthesia was administered and supplemented with intravenous sedation when needed. At first, a renal artery digital subtraction angiography was performed, and the operator determined the degree of stenosis based on the angiography images. In terms of diameter stenosis, less than 50% was considered mild, 50–70% moderate, 70–99% severe, and 100% occlusion. For the patients with severe stenoses and occlusions, endovascular treatments were performed. A 7F short sheath was exchanged for artery access, a 7F guiding catheter was used to engage the ostium of the stenotic renal artery, and a 0.014-inch guidewire was manipulated to cross the stenotic lesion. A rapid exchange balloon, 3–5 mm in diameter, was advanced along the guidewire to predilate the lesion. After predilation, a balloon-expandable stent was induced through the guiding catheter and deployed at the stenotic segment. The length and diameter of the balloons and stents were chosen at the discretion of the operator. Completion angiography was performed to confirm procedural success. Technical success was defined as a <30% residual stenosis at the completion angiography.

### Antiplatelet and Antihypertensive Protocol

Before the procedure, all patients underwent dual antiplatelet therapy (aspirin 100 mg/day and clopidogrel 75 mg/day) for at least 7 days or took a loading dose (aspirin 300 mg and clopidogrel 300 mg) 6 h before the procedure. During the procedure, heparin (3000 IU) was administered for anticoagulation after the insertion of a guiding catheter. All patients were prescribed dual antiplatelet therapy (aspirin 100 mg/day and clopidogrel 75 mg/day) for at least 3 months after the procedure and were converted to a single agent after that. Statin was prescribed for patients with dyslipidemia. Antihypertensive drugs were kept unchanged at baseline and after the endovascular treatment.

### Magnetic Resonance Imaging Scan Protocol

All BOLD-MRI examinations were performed with a MAGNETOM Aera 1.5T MR scanner (Siemens Healthcare GmbH, Erlangen, Germany) using an 18-channel body coil combined with a 32-channel spine coil. The BOLD-MRI examination was a plain scan without the use of contrast agents. A coronal position protocol of multiple-echo spoiled gradient recalled echo through the center of both kidneys was prescribed. The parameters were as follows: repetition time, 130 msec; 5 echoes (echo times: 9.43, 14.29, 19.05, 23.81, and 28.30 msec); flip angle, 90°; slices, 4; section thickness, 6 mm; slice gap, 1.8 mm; imaging matrix, 256 × 192; and field of view, 380 mm × 297 mm. The scanning sequence automatically calculated and produced the T2*-weighted mapping images.

### Magnetic Resonance Imaging Quality Evaluation and Data Analysis

Two radiologists independently assessed the image quality of the BOLD-MRI in terms of image clarity, artifacts, and geometric distortion. Image quality was rated according to a 5-point Likert-type scale (1, non-diagnostic; 2, poor; 3, moderate; 4, good; and 5, excellent).

**FIGURE 1:**
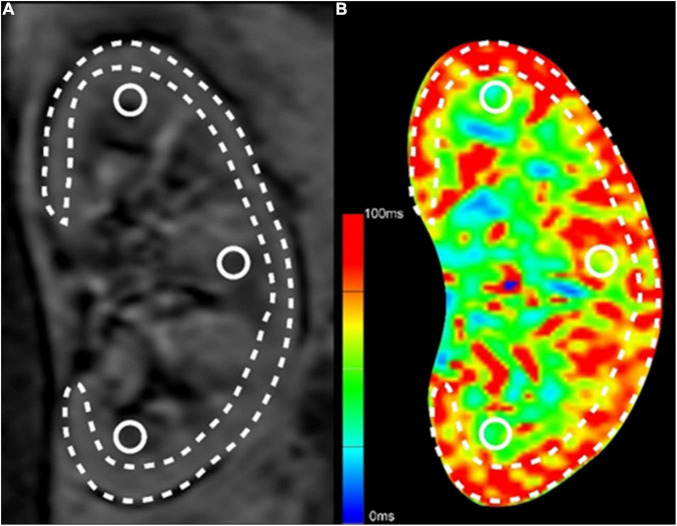
Methods for ROI selection on BOLD-MRI images. **(A)** The original T2*-weighted image; **(B)** The pseudo-color T2*-weighted image. The long and narrow area depicted by the dashed line was the ROI of renal cortex, excluding the renal medulla, collecting system, incidental cysts, and hilar vessels. The three circular areas on the upper, middle and lower medulla of the kidney were the ROIs of the medulla.

Analysis of BOLD-MRI data from coronal position images was performed by drawing a parenchymal region of interest (ROI) on the sections through each kidney’s mid pole hilar region on the T2*-weighted mapping images. Four ROIs were traced: One ROI of cortex was calculated after manual segmentation of the cortex while paying attention excluding the renal medulla, collecting system, incidental cysts, and hilar structures; three ROIs of the medulla were placed in circular areas on the upper, middle, and lower medulla of the kidney, with a diameter of about 3 mm (Figure 1).

After being trained in the standard unified measurement process, two radiologists measured the T2* values of the BOLD-MRI images separately in a blinded fashion. Any discrepancies were resolved in a consensus reading. The final T2* values were the average of the data measured by the two radiologists.

One T2* value was obtained for the cortex of each kidney. Three T2* values of ROIs were obtained for the medulla, and the average was calculated. Finally, to evaluate the degree of hypoxia in the kidney tissue, these values were converted into R2* values according to the following formula: R2* = 1/T2*.

### Follow-Up and Definitions

Follow-up visits were scheduled at 1-month after discharge at the outpatient department. During the follow-up appointment, symptom inquiry, physical examination, renal artery duplex ultrasound scanning and nuclear renal dynamic imaging were conducted. The renal artery ultrasound measurements included peak systolic velocity (PSV) and RI. In the nuclear renal dynamic imaging scan, a bolus of 99mTc-DTPA (Technetium-99m diethylene triamine penta-acetic acid) was given through an antecubital vein. For renal blood flow evaluation, serial dynamic images are acquired every 2 s for a minute. To evaluate renal parenchymal function, a series of images are obtained after 1 min of the renal perfusion phase. Images are acquired at 60 s intervals for a period of 20 min.

Loss of patency was defined as ≥50% restenosis or stent thrombosis based on duplex ultrasound scanning. Change of GFR was defined as the difference between the GFR taken at the 1-month follow-up and the baseline. A modified definition of SRF improvement was the change of GFR was ≥15% of the baseline GFR of the affected kidney and the absolute improvement was at least 5 ml/min.

### Statistical Analysis

Statistical analysis was performed using SPSS software (version 20.0, IBM Corp., Armonk, NY, United States). The normally distributed continuous data are shown as mean ± standard deviation, continuous data without normal distribution are shown as the median and interquartile, and categorical data are shown as numbers and percentages. The normally distributed paired continuous variables were compared by 2-sided Student’s *t*-tests, and the non-normally distributed paired continuous variables were compared by the Wilcoxon signed-rank test. Mean values of independent variables were compared using an independent samples *t*-test when the homogeneity of variance was present and using Welch’s *t*-test when the variance homogeneity was absent. Categorical variables were compared using the 2-sided likelihood ratio chi-square test or Fisher exact test.

Pearson correlation coefficient was used to evaluate the correlation between the two groups of data with normal distribution, and Spearman correlation coefficient was used to evaluate the correlation between the two groups of data without normal distribution. Furthermore, 0.4 ≤ |r|< 0.7 was considered a moderate correlation, 0.7 ≤ |r|< 0.9 a high correlation, and |r| ≥ 0.9 a very high correlation.

The kappa value was used to analyze image quality consistency through a grade score, and the intraclass correlation coefficient (ICC) was used to evaluate the consistency of data measurement. The consistency was reported as poor when ICC <0.4, good when 0.4 ≤ ICC ≤ 0.75, and very good when ICC >0.75.

Receiver operating characteristic (ROC) curve analysis was performed to evaluate baseline cortical and medullary R2* performance across the patients analyzed. The area under the curve (AUC) was used to assess the overall accuracy. Youden’s index and clinical relevance would be considered when selecting the optimum cut-off point. Univariate and multivariate logistic regression were used to analyze the risk factors of the improvement of SRF after RAS. Characteristics of patients and lesions, and BOLD parameters were placed in the analysis model.

Outcomes were reported as the hazard ratio and 95% confidence interval (CI). A *P*-value of less than 0.005 was considered statistically significant.

## Results

### Patient and Procedural Characteristics

**FIGURE 2:**
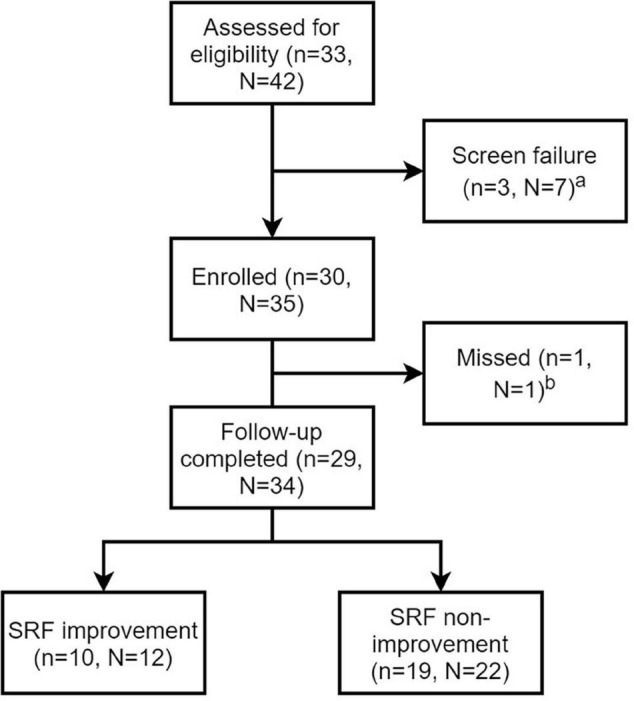
Flow chart of patients and lesions. n stands for number of patients and N stands for number of renal artery lesions. *^a^*Screen failure was due to <70% stenosis confirmed by angiography in three patients with unilateral renal artery stenosis and unsuccessful guidewire crossing in four patients with bilateral renal artery lesions. *^b^*The missing patient had 1-month follow-up, but didn’t complete the nuclear renal dynamic imaging.

A total of 30 patients with ARAS were included in this study. The flowchart of patients is shown in Figure 2. Demographic and baseline information is shown in Table 1. The average age was 63.97 ± 9.44 years, and 73.3% of patients were male. All patients suffered from high blood pressure, and the median number of antihypertensive agents was 2.

**TABLE 1:** Patient characteristics (*n* = 30).

Characteristics	*N* = 30
Age, years	63.97 ± 9.44
Male, *n* (%)	22 (73.30)
BMI (kg/m^2^)	25.00 ± 2.71
Diabetes, *n* (%)	8 (26.70)
Dyslipidemia, *n* (%)	17 (56.70)
Cerebrovascular disease, *n* (%)	6 (20.00)
Coronary heart disease, *n* (%)	12 (40.00)
Peripheral artery disease, *n* (%)	2 (6.70)
Smoking, *n* (%)	14 (46.7)
Hypertension, *n* (%)	30 (100.00)
Duration of hypertension, years	4 (1.00,10.00)
Systolic blood pressure (mmHg)	160.77 ± 20.20
Diastolic blood pressure (mmHg)	87.70 ± 11.56
Kinds of antihypertensive agents	2 (1.00, 3.00)
Use of ACEI/ARB, *n* (%)	12 (40.00)
Use of diuretics, *n* (%)	7 (23.30)
Use of β-Blockers	12 (40.00)
Renal insufficiency, *n* (%)	11 (36.67)
Serum creatinine (μmol/L)	125.75 (97.06, 147.94)
eGFR (mL/min/1.73m^2^)	52.64 ± 18.72

*BMI, Body Mass Index; ACEI, Angiotensin Converting-Enzyme Inhibitor; ARB, Angiotensin II Receptor Blocker; eGFR, estimated Glomerular Filtration Rate; Renal insufficiency was defined as serum creatinine >133 μmol/L.*

Bilateral renal artery lesions were found in 9 patients, 4 of whom had unilateral renal artery occlusion that could not be recanalized successfully, and unilateral ARAS was found in 21 patients who all undertook RAS successfully. Overall, 35 renal arteries were treated by stents (31 PALMAZ BLUE, Cordis, FL, United States; 1 Herculink Elite, Abbott Vascular, CA, United States;1 Acculink, Abbott Vascular, CA, United States and 2 Express Vascular, Boston Scientific, MA, United States).

### Quality and Consistency of Blood Oxygen Level-Dependent Imaging

The quality of the acquired images met the need for diagnosis regarding resolution, artifacts, and deformation. The kappa value of the image quality was 0.816.

A total of 35 kidneys with severe ARAS were included for evaluation of cortical and medullary T2* at baseline. The ICC was 0.921 (95% CI 0.887–0.945) for cortical T2* and 0.818 (95% CI 0.74–0.871) for medullary T2*, indicating that the two radiologists noted a high consistency in estimating both cortical and medullary T2*.

### Oxygenation Level of Kidneys With Severe Atherosclerotic Renal Artery Stenosis

The oxygenation level of affected kidneys was compared with the contralateral kidneys in the 21 patients with severe unilateral ARAS. The cortical and medullary T2* values of the affected kidneys were significantly lower than those of the contralateral kidneys (*P* = 0.007 and *P* < 0.001, respectively). Accordingly, the R2* (1/T2*) values were higher in the affected side than in the contralateral side (*P* = 0.003 and *P* < 0.001, respectively). Details are shown in Table 2.

**TABLE 2:** BOLD-MRI comparison of the affected kidney and contralateral kidney in patients with unilateral ARAS.

		The severely stenotic kidney (*n* = 21)	The contralateral kidney (*n* = 21)	*P*
Cortical	T2* (ms)	78.12 ± 10.86	85.16 ± 5.50	0.007
	R2* (s^–1^)	13.03 ± 1.78	11.79 ± 0.74	0.003
Medullary	T2* (ms)	38.37 ± 4.96	44.16 ± 2.59	<0.001
	R2* (s^–1^)	26.48 ± 3.38	22.72 ± 1.35	<0.001

*BOLD-MRI, Blood Oxygen Level-Dependent Magnetic Resonance Imaging; ARAS, Atherosclerotic Renal Artery Stenosis.*

### One-Month Outcomes of Endovascular Treatment

All patients with severe ARAS had a 1-month follow-up. The parameters of global renal function and blood pressure at both the baseline and follow-up are given in Table 3. The average systolic blood pressure decreased from 160.77 ± 20.20 mmHg at baseline to 132.80 ± 10.56 mmHg at the 1-month follow-up (*P* < 0.001). The average diastolic blood pressure decreased from 87.70 ± 11.56 mmHg to 78.27 ± 9.00 mmHg (*P* < 0.001). Parameters regarding global renal function did not change.

**TABLE 3:** Comparison of global renal function and blood pressure at baseline and 1-month follow-up.

Variables	At baseline (*n* = 30)	At 1-month follow up (*n* = 30)	*P*
Serum creatinine (μmol/L)	125.75 (97.06, 147.94)	118.50 (97.43, 146.25)	0.951
eGFR (mL/min/1.73m^2^)	52.64 ± 18.72	53.39 ± 17.16	0.695
Urine creatinine (mmol/L)	4.70 (3.85, 7.45)	7.05 (4.78, 12.40)	0.063
Urine microalbumin (mg/L)	27.90 (3.91,166.50)	22.35 (6.23, 63.70)	0.370
Systolic blood pressure (mmHg)	160.77 ± 20.20	132.80 ± 10.56	<0.001
Diastolic blood pressure (mmHg)	87.70 ± 11.56	78.27 ± 9.00	<0.001

*eGFR, estimated Glomerular Filtration Rate.*

For the 35 renal arteries treated and evaluated at follow-up, no loss of patency was found by ultrasound (Table 4). The velocity of renal artery flow decreased significantly (0.98 ± 0.34 m/s vs. 2.96 ± 1.45 m/s, *P* < 0.001). One patient did not complete the nuclear renal dynamic imaging at the 1-month follow-up. SRF improvement occurred in 35.3% (12/34) of the stented kidneys in 34.5% (10/29) of the patients. The average split GFR increased significantly (19.56 ± 11.86 ml/min vs. 17.16 ± 11.69 ml/min, *P* = 0.013).

**TABLE 4:** The nuclear renal dynamic and Doppler ultrasound parameters of stented kidneys at baseline and 1-month follow-up (*n* = 35).

Parameters	Baseline	1 month	*P*-value
GFR (ml/min)	17.16 ± 11.69	19.56 ± 11.86	0.013
PSV (m/s)	2.96 ± 1.45	0.98 ± 0.34	<0.001
RI	0.69 ± 0.13	0.71 ± 0.09	0.298

*GFR, Glomerular Filtration Rate; PSV, Peak Systolic Velocity; RI, Resistance Index.*

### The Correlation of Oxygenation Level at Baseline and Change of Glomerular Filtration Rate

**FIGURE 3:**
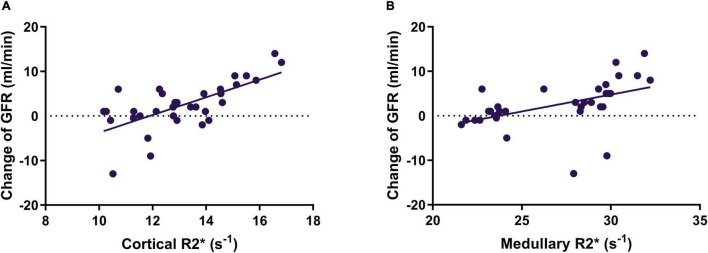
The correlation between cortical R2* **(A)** and medullary R2* **(B)** and change of SRF of the stented kidney. SRF, split renal function; GFR, glomerular filtration rate.

Correlations between the baseline oxygenation level of the renal cortex and medulla, and the 1-month change of ipsilateral GFR were analyzed in the 34 kidneys treated by RAS. Pearson correlation analysis showed that there was a moderate positive correlation between the GFR change and baseline cortical R2* value (*r* = 0.674; *P* < 0.001; Figure 3A) and baseline medullary R2* value (*r* = 0.483; *P* = 0.004; Figure 3B).

The baseline cortical R2* and medullary R2* in the SRF improvement group (14.4 ± 1.8 s^–1^ and 29.5 ± 2.6 s^–1^, respectively) were higher (*P* = 0.001 and *P* < 0.001, respectively) than those in the non-improvement group (12.4 ± 1.3s^–1^ and 25.4 ± 2.9 s^–1^, respectively), indicating that baseline cortical R2* and medullary R2* values could be candidates for predicting SRF improvement. For patients with SRF improvement, the serum creatinine level and eGFR did not change significantly (*P* = 0.321 and *P* = 0.228).

### The Correlation of Oxygenation Level at Baseline and Change of Blood Pressure

The correlations of BOLD-derived renal oxygenation level and evolution of blood pressure were analyzed in 25 patients treated by RAS unilaterally. The baseline medullary R2* had no correlation with absolute systolic blood pressure (SBP) change (*r* = 0.102 and *P* = 0.628), relative SBP change (*r* = 0.138 and *P* = 0.509), absolute diastolic blood pressure (DBP) change (*r* = 0.195 and *P* = 0.352), and relative DBP change (*r* = 0.265 and *P* = 0.200). Similarly, no correlation was found between the baseline cortical R2* and changes of blood pressure (absolute SBP *P* = 0.289; relative SBP *P* = 0.308; absolute DBP *P* = 0.494; relative DBP *P* = 0.484).

### The Role of R2* at Baseline in Predicting Split Renal Function Improvement

**FIGURE 4:**
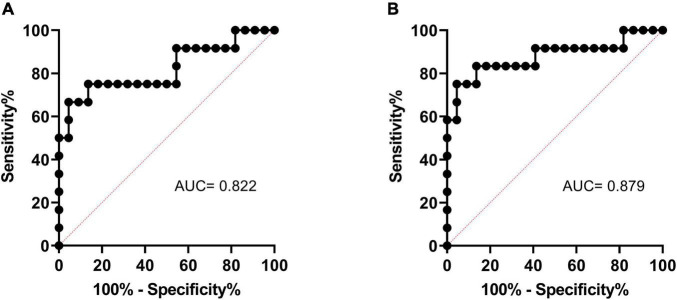
The ROC curve of cortical R2* **(A)** and medullary R2* **(B)** in predicting SRF improvement. ROC, receiver operating characteristic; SRF, split renal function.

The ROC curves of cortical R2* and medullary R2* are shown in Figure 4. The AUC of medullary R2* was 0.879 (95% CI 0.736–1.000), larger than that of cortical R2* (0.822, 95% CI 0.655–0.989), indicating that medullary R2* had higher accuracy and is a better predictor. The cut-off value of baseline medullary R2* was 29.1 s^–1^, the sensitivity was 0.833 (95% CI 0.552–0.970), and the specificity was 0.864 (95% CI 0.667–0.953). The absolute values of GFR change were higher in patients with medullary R2* > 29.1 s^–1^ than in patients with medullary R2* ≤ 29.1 s^–1^ (5.769 ± 5.644 vs. 0.309 ± 3.945, *P* = 0.002). In the univariable model (Table 5), only cortical and medullary R2* were significantly associated with SRF improvement. Since cortical and medullary R2* were linearly correlated (*r* = 0.584, *P* < 0.001) and PSV was considered clinically relevant, only medullary R2* and PSV were placed in the multivariable model. Medullary R2* was the only independent predictor of SRF improvement (*P* = 0.034, OR 3.017, 95%CI 1.089–8.358).

**TABLE 5:** Univariable and Multivariable predictors of split renal function improvement after renal artery stenting.

	Univariable analysis				Multivariable analysis			
Predictors	*p*-value	OR	95% confidence interval		*p*-value	OR	95% confidence interval	
Age	0.462	1.031	0.951	1.117				
Male	0.881	1.133	0.219	5.864				
Smoking	1.000	1.000	0.245	4.083				
Duration of hypertension	0.133	0.883	0.750	1.039				
Diabetes	0.346	2.333	0.400	13.609				
Dyslipidemia	0.611	1.444	0.351	5.947				
Stroke	0.881	0.882	0.171	4.565				
Coronary artery disease	0.966	0.969	0.232	4.042				
Baseline GFR	0.878	0.995	0.936	1.058				
Degree of stenosis	0.641	1.023	0.930	1.124				
Baseline PSV	0.217	1.402	0.820	2.396	0.549	0.798	0.381	1.671
Baseline RI	0.510	0.163	0.001	36.047				
Baseline kidney length	0.353	1.352	0.715	2.554				
Cortical R2*	0.006	2.419	1.290	4.539				
Medullary R2*	0.006	1.674	1.157	2.422	0.034	3.017	1.089	8.358

*GFR, Glomerular Filtration Rate; PSV, Peak Systolic Velocity; RI, Resistance Index.*

## Discussion

This pilot study elucidated that the R2* value of BOLD-MRI was an effective non-invasive biomarker for selecting ARAS patients who might benefit from RAS in SRF improvement. The AUC of medullary R2* was 0.879 (95% CI 0.736–1.000), the sensitivity was 0.833 (95% CI 0.552–0.970), and the specificity was 0.864 (95% CI 0.667–0.953) if the medullary R2* was ≥29.1 s^–1^.

Blood oxygen level-dependent magnetic resonance imaging is a non-invasive technique for evaluating kidney tissue oxygenation without requiring contrast exposure (14). A normally perfused kidney would be expected to have a low R2* score in the cortex and a higher R2* score in the deeper medullary region, consistent with the known hypoxia gradient in deep medullary areas (9). A low R2* score is observed if there is adequate perfusion and tissue function, but it can also be seen if the kidney is non-functioning, as the oxygenated blood passes through without oxygen utilization or consumption by atrophic tissue (11, 13). Therefore, high R2* appears when there is a significant decrease in renal blood flow and the kidney is still salvageable, which is exactly the condition in which ASAS patients may benefit from RAS. In the present study, the AUC of medullary R2* was 0.879, indicating high accuracy in predicting SRF improvement after RAS.

The related randomized trials may have been confined by the use of global renal function as the outcome assessment (4–6). Due to the compensation achieved by hyperfiltering of the contralateral kidney in unilateral severe ARAS patients, there may be no net decline detected in the global renal function (9, 15, 16). In the present study, nuclear dynamic imaging was used to assess the SRF improvement of the stented kidney. In the SRF improvement group, the global renal function evaluated by serum creatinine did not improve significantly, which supports the theory mentioned above (9). Blood pressure improvement is an important target for treating ARAS, and its predictors, including translesional pressure gradients, intravascular ultrasound, and angiography, have been previously investigated and compared (17). In the present study, the blood pressure improvement was not correlated with baseline cortical and medullary R2*. The possible mechanism is that blood pressure is associated with hemodynamic disturbance rather than the hypoxic condition.

The potential limitations of our study should be noted. First, SRF improvement was only evaluated at 1-month follow-up, and therefore long-term results remain unknown. The reason for the relatively short follow-up is that other comorbidities that ARAS patients have can adversely affect renal function. For this reason, it is difficult to determine whether RAS improves renal function in the long run. Second, the study was confined to a single center, and the bias associated with the sample size may limit the generalizability of the results. Third, the BOLD-derived oxygen levels cannot predict the outcomes of total GFR and blood pressure improvement, which limits the clinical impact.

## Conclusion

Blood oxygen level-dependent magnetic resonance imaging is useful for identifying severe ARAS patients whose SRF could benefit from RAS. We found medullary R2* ≥ 29.1 s^–1^ to have good sensitivity and specificity, making it an excellent predictor of SRF improvement after RAS.

## Data Availability Statement

The raw data supporting the conclusions of this article will be made available by the authors, without undue reservation.

## Ethics Statement

The studies involving human participants were reviewed and approved by Peking University First Hospital Ethics Committee. The patients/participants provided their written informed consent to participate in this study.

## Author Contributions

ZL, BZ, and LL contributed in implementing and writing. RW, GN, and ZY contributed in implementing and data analysis. YZ, XT, JQ, and MY contributed in supervising. All authors contributed to the article and approved the submitted version.

## Conflict of Interest

The authors declare that the research was conducted in the absence of any commercial or financial relationships that could be construed as a potential conflict of interest.

## Publisher’s Note

All claims expressed in this article are solely those of the authors and do not necessarily represent those of their affiliated organizations, or those of the publisher, the editors and the reviewers. Any product that may be evaluated in this article, or claim that may be made by its manufacturer, is not guaranteed or endorsed by the publisher.
